# Dual EZH2 and G9a inhibition suppresses multiple myeloma cell proliferation by regulating the interferon signal and IRF4-MYC axis

**DOI:** 10.1038/s41420-020-00400-0

**Published:** 2021-01-12

**Authors:** Kazuya Ishiguro, Hiroshi Kitajima, Takeshi Niinuma, Reo Maruyama, Naotaka Nishiyama, Hitoshi Ohtani, Gota Sudo, Mutsumi Toyota, Hajime Sasaki, Eiichiro Yamamoto, Masahiro Kai, Hiroshi Nakase, Hiromu Suzuki

**Affiliations:** 1 Department of Molecular Biology, Sapporo Medical University School of Medicine, Sapporo, Japan; 2 Department of Gastroenterology and Hepatology, Sapporo Medical University School of Medicine, Sapporo, Japan; 3 Project for Cancer Epigenomics, Cancer Institute, Japanese Foundation for Cancer Research, Tokyo, Japan; 4 Department of Urology, Graduate School of Medicine and Pharmaceutical Sciences for Research University of Toyama, Toyama, Japan; 5 Laboratory of Genome and Epigenome Dynamics, Department of Animal Sciences, Graduate School of Bioagricultural Sciences, Nagoya University, Nagoya, Japan

**Keywords:** Myeloma, Targeted therapies

## Abstract

Epigenetic mechanisms such as histone modification play key roles in the pathogenesis of multiple myeloma (MM). We previously showed that EZH2, a histone H3 lysine 27 (H3K27) methyltransferase, and G9, a H3K9 methyltransferase, are potential therapeutic targets in MM. Moreover, recent studies suggest EZH2 and G9a cooperate to regulate gene expression. We therefore evaluated the antitumor effect of dual EZH2 and G9a inhibition in MM. A combination of an EZH2 inhibitor and a G9a inhibitor strongly suppressed MM cell proliferation in vitro by inducing cell cycle arrest and apoptosis. Dual EZH2/G9a inhibition also suppressed xenograft formation by MM cells in vivo. In datasets from the Gene Expression Omnibus, higher *EZH2* and *EHMT2* (encoding G9a) expression was significantly associated with poorer prognoses in MM patients. Microarray analysis revealed that EZH2/G9a inhibition significantly upregulated interferon (IFN)-stimulated genes and suppressed IRF4-MYC axis genes in MM cells. Notably, dual EZH2/G9a inhibition reduced H3K27/H3K9 methylation levels in MM cells and increased expression of endogenous retrovirus (ERV) genes, which suggests that activation of ERV genes may induce the IFN response. These results suggest that dual targeting of EZH2 and G9a may be an effective therapeutic strategy for MM.

## Introduction

Multiple myeloma (MM) is an incurable disorder caused by monoclonal proliferation of abnormal plasma cells, resulting in hypercalcemia, renal failure, anemia, and bone lesions. MM accounts for 1.8% of all cancers and 18% of blood cancers, and will cause an estimated 12,830 deaths in the United States in 2020^1^. Because immune surveillance is disrupted in MM, immunotherapies, including immunomodulatory drugs (IMiDs; thalidomide, lenalidomide, and pomalidomide) and monoclonal antibodies (elotuzumab and daratumumab), are the primary approach to treatment of MM. In addition, the effectiveness of immune check point inhibitors such as nivolumab and pembrolizumab has also been tested in a number of clinical trials^2^.

Evidence now suggests that epigenetic alterations, including aberrant DNA methylation and histone modifications, are involved in the pathogenesis of MM and that their plasticity makes them promising therapeutic targets^3^. For example, when combined with bortezomib and dexamethasone, the histone deacetylase (HDAC) inhibitor panobinostat is reportedly effective in patients with relapsed/refractory MM^4^. In addition, the efficacies against MM of a number of epigenetic drugs, including a DNA methyltransferase (DNMT) inhibitor (Azacitidine), a BET inhibitor (CPI-0610), and HDAC inhibitors (Vorinostat, Belinostat and CI-994), as well as a combination of DNMT and HDAC inhibitors (Azacitidine plus phenylbutyrate) have been tested in clinical trials^3,5^.

Histone methylation plays essential roles in the regulation of gene expression. For instance, methylation of histone H3 lysine 4 (H3K4), histone H3 lysine 36 (H3K36), and histone H3 lysine 79 (H3K79) is generally associated with transcriptional activation, while methylation of histone H3 lysine 9 (H3K9), histone H3 lysine 27 (H3K27) and histone H4 lysine 20 (H4K20) is associated with gene silencing^6^. Dysregulation of histone methylation is deeply involved in the pathogenesis of MM, and recent preclinical studies have demonstrated the antimyeloma effects of inhibitors of EZH2, a H3K27 methyltransferase^7–9^, and DOT1L, a H3K79 methyltransferase^10,11^. Indeed, as a result of its promising efficacy against MM, patients with relapsed or refractory MM were recently recruited for a phase I clinical trial of the EZH2 inhibitor, GSK126 (also known as GSK2816126)^12^.

We recently evaluated the antimyeloma effects of a series of inhibitors of histone methylation modifiers, and found that both EZH2 and a H3K9 methyltransferase, G9a, are potential therapeutic targets in MM^10^. Recent studies showed that EZH2 and G9a act cooperatively to suppress gene expression in mouse embryonic stem cells and human fibroblasts^13,14^. In breast cancer cells, an epigenetic factor, CDYL2, recruits EZH2 and G9a to repress expression of the tumor suppressive microRNA gene *MIR124* and to promote cancer cell migration, invasion, and stemness^15^. These reports suggest that dual targeting of EZH2 and G9a may be an effective cancer treatment strategy, although crosstalk between EZH2 and G9a has not been studied in MM. In the present study, we evaluated the effectiveness of single and dual inhibition of these histone methyltransferases in MM.

## Results

### Dual inhibition of EZH2 and G9a exerts a strong antitumor effect in MM

**Fig. 1:**
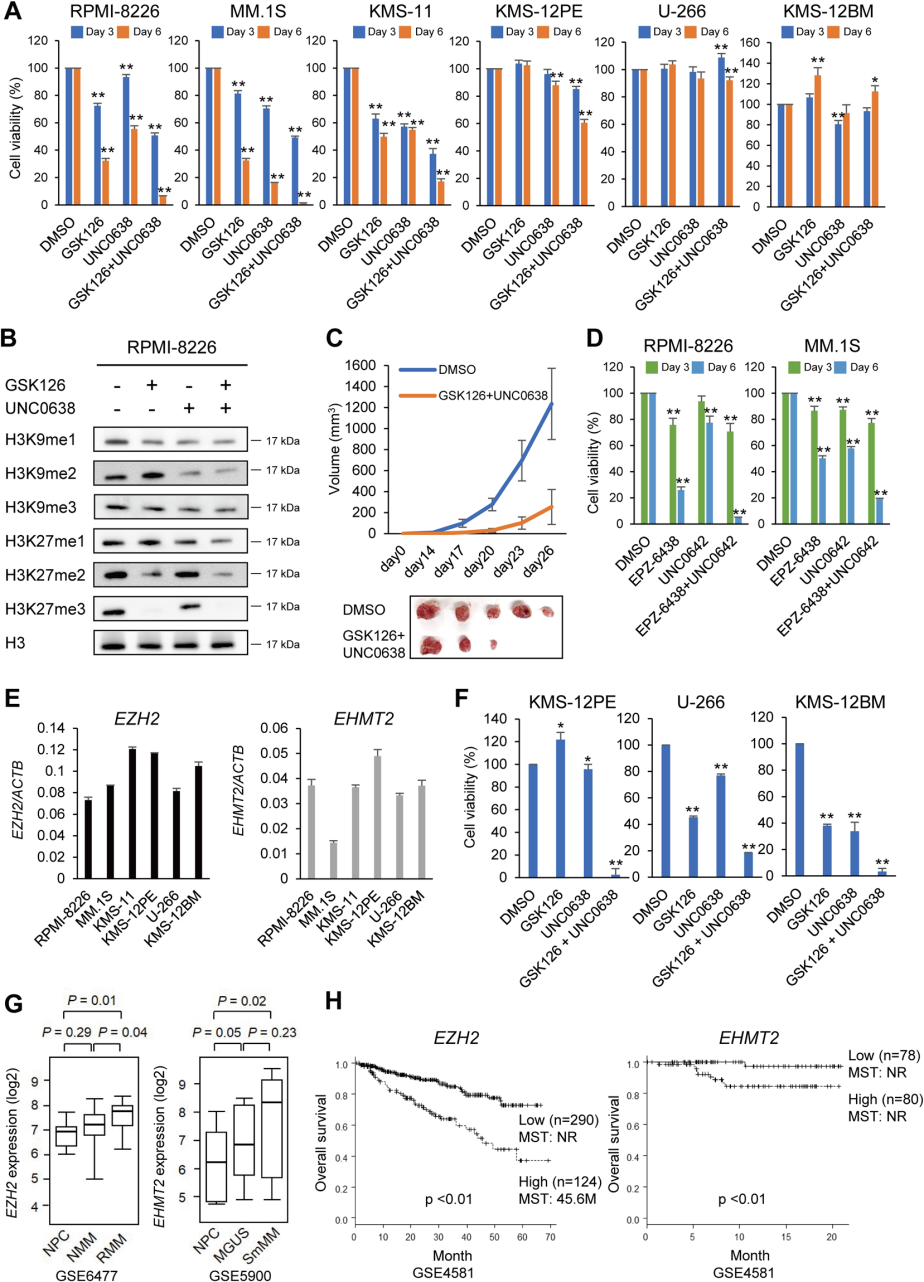
Antitumor effects of EZH2 and G9a inhibition in MM. **A** Effects of an EZH2 inhibitor (GSK126) and/or a G9a inhibitor (UNC0638) on MM cell viability. Shown are summarized cell viability assays in MM cell lines treated with the indicated drugs (1 μM each) for 3 or 6 days. Results are normalized to cells treated with DMSO. The data are presented as means of three biological replications; error bars represent standard errors of the mean (SEMs). **B** Western blot analysis of H3K9 and H3K27 methylation in RPMI-8226 cells treated with the indicated inhibitors (1 μM each, 6 days). **C** Tumor growth in mice injected with RPMI-8226 cells pretreated with DMSO or GSK126 + UNC0638 (1 μM each). Growth curves are presented as means of five biological replications. Resected tumors are shown below. **D** Results of cell viability assays using indicated MM cell lines treated with an EZH2 inhibitor (EPZ-6438, 1 μM) and/or a G9a inhibitor (UNC0642, 1 μM). Shown are means of three biological replications; error bars represent SEMs. **E** qRT-PCR analysis of *EZH2* and *EHMT2* in the indicated MM cell lines. Results are normalized to *ACTB* expression. Shown are means of three technical replications; error bars represent SEMs. **F** Results of cell viability assays of MM cell lines treated for 14 days with the indicated drugs (1 μM each). Shown are means of five biological replications; error bars represent SEMs. **G** Comparison of *EZH2* mRNA expression among normal plasma cells (NPC, *n* = 15), newly diagnosed MM (NMM, *n* = 69) and relapsed MM (RMM, *n* = 28) (left), and *EHMT2* mRNA expression among normal plasma cells (NPC, *n* = 22), monoclonal gammopathy of undetermined significance (MGUS, *n* = 44) and smoldering multiple myeloma (SmMM, *n* = 12) (right) using the indicated datasets. **H** Kaplan–Meier curves showing the effect of *EZH2* or *EHMT2* expression on overall survival of newly diagnosed MM patients from the indicated datasets. MST mean survival time, NR not reached. *P < 0.05, **P < 0.01.

To determine whether EZH2 and G9a could be potential therapeutic targets in MM, we first treated 6 MM cell lines with the EZH2 inhibitor GSK126 (1 μM), the G9a inhibitor UNC0638 (1 μM), or GSK126 + UNC0638 (1 μM each) for 3 or 6 days (1 μM) (Fig. 1A). Subsequent cell viability assays revealed that treatment with the respective agents moderately suppressed proliferation of three cell lines (RPMI-8226, MM.1S, and KMS-11) in a time dependent manner, while the combination of the two inhibitors exerted stronger effects (Fig. 1A). We observed similar results in KMS-12PE cells, though the response was more limited. The remaining two cell lines (U-266 and KMS-12BM) were resistant to these treatments (Fig. 1A).

Reductions in the levels of histone methylation induced by the drug treatments were confirmed by western blot analyses using antibodies specific for mono-methylated, di-methylated, and tri-methylated H3K9 and H3K27 (H3K9me1/me2/me3 and H3K27me1/me2/me3) in three sensitive cell lines (RPMI-8226, MM.1S, and KMS-11). (Fig. 1B and Supplementary Fig. S1A). With those drug treatments, we also confirmed reductions of H3K9me2 and H3K27me3 in two resistant cell lines (KMS-12PE and KMS-12BM) (Supplementary Fig. S1B). In addition, ex vivo treatment with GSK126 + UNC0638 significantly diminished xenograft formation by MM cells in SCID mice (Fig. 1C). To confirm the specificity of the drug targets, we treated MM cells with another set of inhibitors against EZH2 (EPZ-6438) and G9a (UNC0642). Again, the combination of the two agents (1 μM each) exerted stronger antiproliferative effects than either agent alone (Fig. 1D).

To clarify whether levels of EZH2 and G9a expression are associated with the sensitivity to inhibitors, we performed qRT-PCR with *EZH2* and *EHMT2* (encoding G9a) and western blot analyses. We found that levels of the mRNA and protein expression varied among the MM cell lines and were not consistent with the drug sensitivities (Fig. 1E and Supplementary Fig. S2). Because it takes relatively long times for epigenetic drugs to exert their antitumor effects, we next treated the resistant cell lines (KMS-12PE, U-266, and KMS-12BM) with GSK126 and UNC0638 for 14 days. The extended treatment significantly enhanced the antiproliferative effects, and dual inhibition of EZH2 and G9a exerted stronger effects than inhibition of either enzyme alone (Fig. 1F). These findings suggest that the sensitivities of MM cells to the dual inhibition vary among cell lines, and that the antiproliferative effect is generally time-dependent.

Analysis using datasets from the Gene Expression Omnibus revealed that *EZH2* expression levels were significantly higher in relapsed MM (RMM) patients than in normal plasma cells (NPCs) or newly diagnosed MM (NMM) patients (Fig. 1G). Levels of *EHMT2* expression were also higher in monoclonal gammopathy of undetermined significance (MGUS), and smoldering MM (SmMM) patients than in NPCs (Fig. 1G). Moreover, higher levels of *EZH2* or *EHMT2* expression were significantly associated with poorer overall survival in newly diagnosed MM patients (Fig. 1H).

**Fig. 2:**
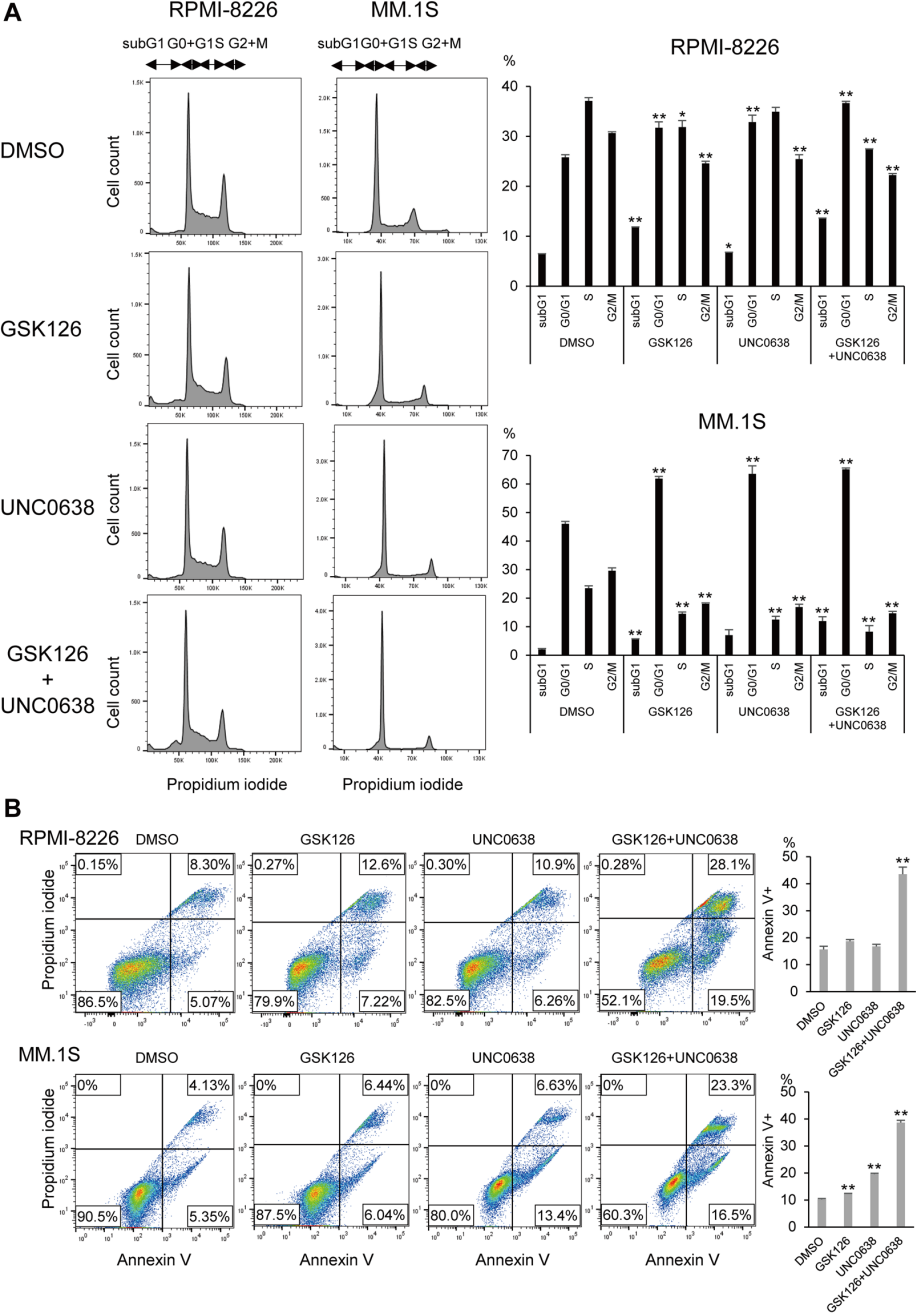
Effects of EZH2 and G9a inhibitors on the cell cycle and apoptosis in MM cells. **A** Cell cycle analysis in MM cell lines treated with the indicated drugs (1 μM, 6 days). Representative data are shown on the left. Summarized data from three biological replications are shown on the right; error bars represent SEMs. **B** Apoptosis assays in MM cells treated with the indicated drugs (1 μM, 6 days). Representative data are shown on the left. Summarized data from three biological replications are shown on the right; error bars represent SEMs. *P < 0.05, **P < 0.01.

Cell cycle analysis revealed that treatment with GSK126 + UNC0638 (1 μM each, 6 days) increased the sub-G1 and G0/G1 phase populations and decreased the S phase population in MM cells (Fig. 2A). In addition, induction of apoptosis by the dual inhibitor treatment was further confirmed by Annexin V staining assays (Fig. 2B). These results suggest that dual inhibition of EZH2 and G9a exerts antimyeloma effects by inducing cell cycle arrest and apoptosis.

### Dual inhibition of EZH2 and G9a activates IFN signaling and blocks the IRF4-MYC axis in MM cells

**Fig. 3:**
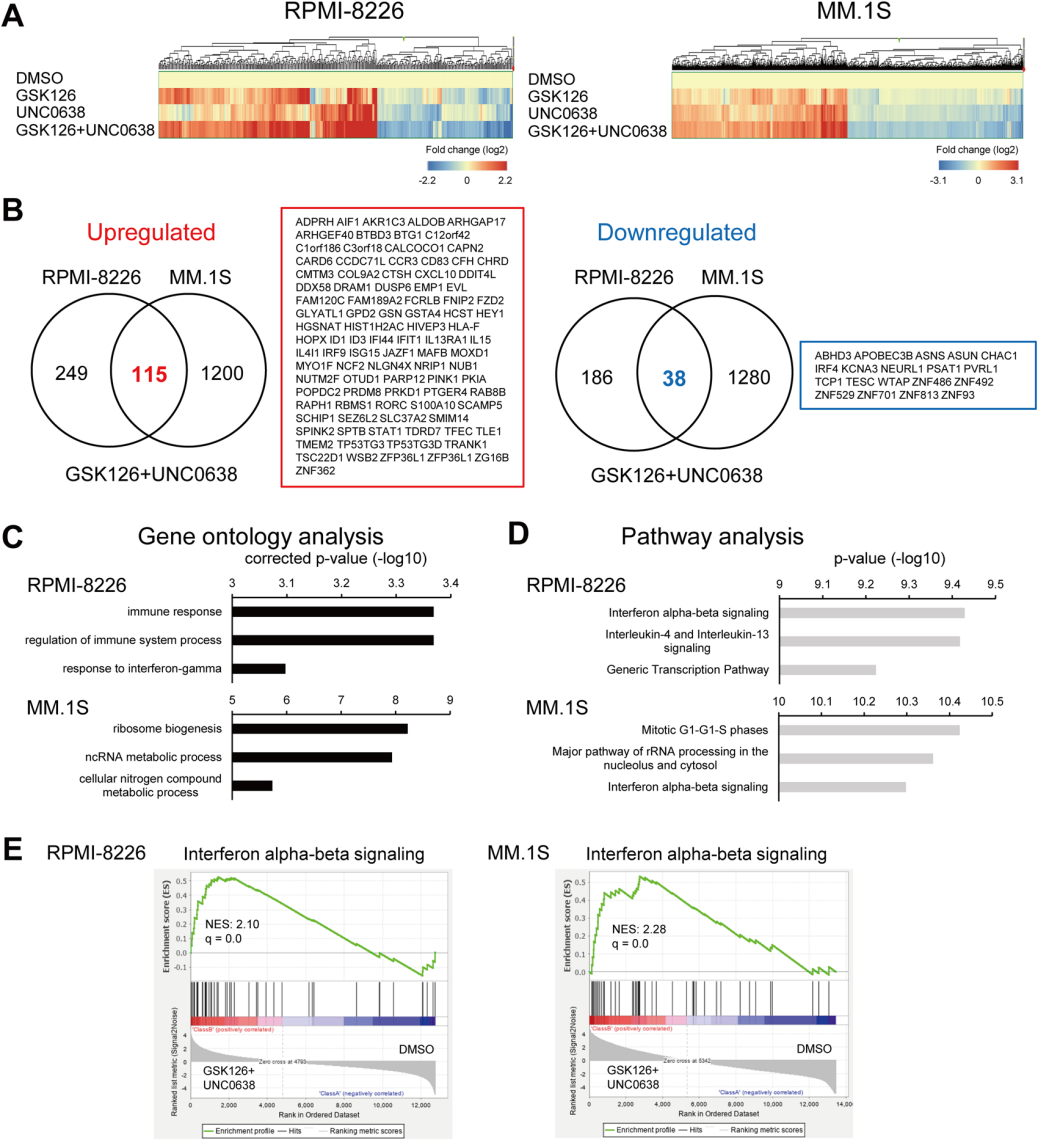
Analysis of gene expression in MM cells after EZH2 and G9a inhibition. **A** Heat maps for the genes whose expression was altered in MM cell lines by the indicated drugs (1 μM each, 6 days). Shown are means of two biological replications. **B** Venn diagrams of genes upregulated (left) or downregulated (right) by GSK126 + UNC0638 in two MM cell lines. Genes upregulated or downregulated in both cell lines are listed in boxes on the right. **C**, **D** Gene ontology (**C**) and pathway analyses (**D**) of genes altered by GSK126 + UNC0638 (>2-fold) in MM cell lines. **E** GSEA of the genes involved in interferon α and β signaling using the microarray data from the indicated MM cell lines treated with DMSO or GSK126 + UNC0638.

To elucidate the mechanism underlying the antimyeloma effects of the dual inhibition of EZH2 and G9a, we performed gene expression microarray analysis using RPMI-8226 and MM.1S cells treated for 6 days with GSK126 (1 μM), UNC0638 (1 μM) or their combination (1 μM each) (Fig. 3A). We found that the effects of the combination treatment on gene expression profiles were greater than those of either agent alone (Fig. 3A and Supplementary Fig. S3). In RPMI-8226 cells, GSK126, UNC0638, and their combination upregulated (>2-fold) 168, 121, and 364 probe sets (151, 104, and 319 genes), while they downregulated (>2-fold) 37, 44, and 224 probe sets (24, 39, and 177 genes). Similar results were observed with MM.1S cells: 332, 573 and 1315 probe sets (301, 488, and 1107 genes) were upregulated (>2-fold), while 55, 262, and 1318 probe sets (44, 203 and 1002 genes) were downregulated (>2-fold) (Supplementary Fig. S3 and Supplementary Tables S2–S13). Among the genes affected by GSK126 + UNC0638, 115 upregulated probe sets (97 genes) and 38 downregulated probe sets (19 genes) were common to the two cell lines (Fig. 3B).

Gene ontology analysis revealed that genes associated with immune and interferon (IFN) responses were significantly enriched among the genes altered by GSK126 + UNC0638 in RPMI-8226 cells (Fig. 3C). Pathway analysis also showed that IFN-α/β signaling was strikingly affected by dual EZH2/G9a inhibition in both cell lines. In addition, genes associated with the cell cycle (G1/G1-S phase) were significantly affected in MM.1S cells (Fig. 3D). The significant effects of the dual EZH2/G9a inhibition on the IFN-α/β signaling genes were further confirmed by GSEA (Fig. 3E). These results suggest that IFN signaling may be associated with the antimyeloma effect of EZH2/G9a inhibition.

**Fig. 4:**
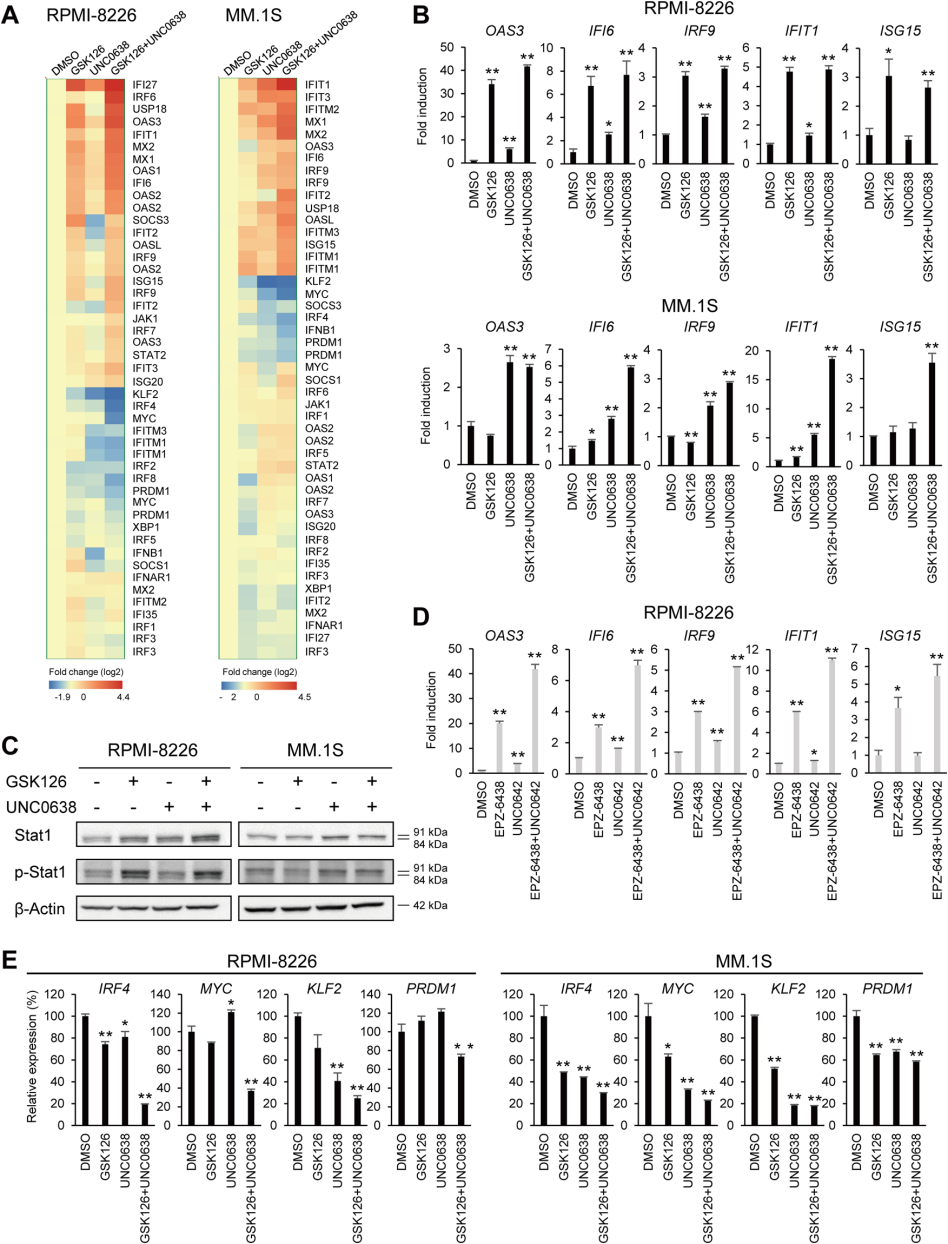
Upregulation of ISGs and suppression of IRF4-MYC axis genes by EZH2 and G9a inhibition in MM cells. **A** Heat maps for expression of ISGs and IRF4-MYC axis genes in MM cell lines treated with the indicated drugs (1 μM each, 6 days). **B** qRT-PCR analysis of ISGs. Results are normalized to cells treated with DMSO. Shown are means of three technical replications; error bars represent SEMs. **C** Western blot analysis of Stat1 and phosphorylated Stat1. **D** qRT-PCR analysis of ISGs in RPMI-8226 cells treated with EPZ-6438 and/or UNC0642. Results are normalized to cells treated with DMSO. Shown are means of three technical replications; error bars represent SEMs. **E** qRT-PCR analysis of IRF4-MYC axis genes. Results are normalized to cells treated with DMSO. Shown are means of three technical replications; error bars represent SEMs. *P < 0.05, **P < 0.01.

The results of a microarray analysis of representative genes are shown in Fig. 4A. Consistent with the bioinformatics analyses, a series of IFN-stimulated genes (ISGs) were upregulated by GSK126 or UNC0638 in both RPMI-8226 and MM.1S cells, and GSK126 + UNC0638 exerted stronger effects than either inhibitor alone. Using qRT-PCR, we confirmed the expression of five ISGs: *OAS3*, *IFI6*, *IRF9*, *IFIT1*, and *ISG15*. In RPMI-8226 cells, these genes were significantly upregulated by GSK126 alone or GSK126 + UNC0638 (Fig. 4B). In MM.1S cells, the combination treatment had the strongest effect on most of the genes analyzed, while UNC0638 also upregulated multiple ISGs (Fig. 4B). Western blot analysis showed that GSK126 + UNC0638 upregulated levels of total and phosphorylated Stat1 in the two cell lines (Fig. 4C). Consistent with the qRT-PCR results, GSK126 or UNC0638 also upregulated total and phosphorylated Stat1 in RPMI-8226 and MM.1S cells, respectively (Fig. 4C). Induction of ISGs was confirmed with another set of inhibitors (EPZ-6438 and UNC0642), and again the combination treatment exerted stronger effects than either single agent (Fig. 4D and Supplementary Fig. S4). These results suggest that the dual inhibition of EZH2 and G9 activates IFN signaling in MM cells.

In contrast to the upregulation of other ISGs, *IRF4* was substantially downregulated by GSK126 + UNC0638 in both RPMI-8226 and MM.1S cells (Figs. 3B and 4A). Survival of MM cells is dependent on IRF4, and we recently showed that inhibition of the histone methyltransferase DOT1L blocks MM cell proliferation by suppressing the IRF4–MYC axis^10,16^. Our microarray data revealed that genes involved in the IRF4–MYC axis (*IRF4*, *MYC*, *KLF2*, and *PRDM1*) were downregulated in cells treated with the inhibitors (Fig. 4A). Moreover, qRT-PCR confirmed that the dual EZH2/G9a inhibition strongly suppressed expression of the four IRF4–MYC axis genes in MM cells, and that treatment with either inhibitor individually also moderately suppressed their expression (Fig. 4E). Similarly, a combination of a second set of inhibitors (EPZ-6438 and UNC0642) also significantly downregulated these genes (Supplementary Fig. S5). Overall, our results suggest that dual inhibition of EZH2 and G9a exerts antimyeloma effects by activating IFN signaling and blocking the IRF4–MYC axis.

### Dual inhibition of EZH2 and G9a reactivates ERV genes in MM cells

**Fig. 5:**
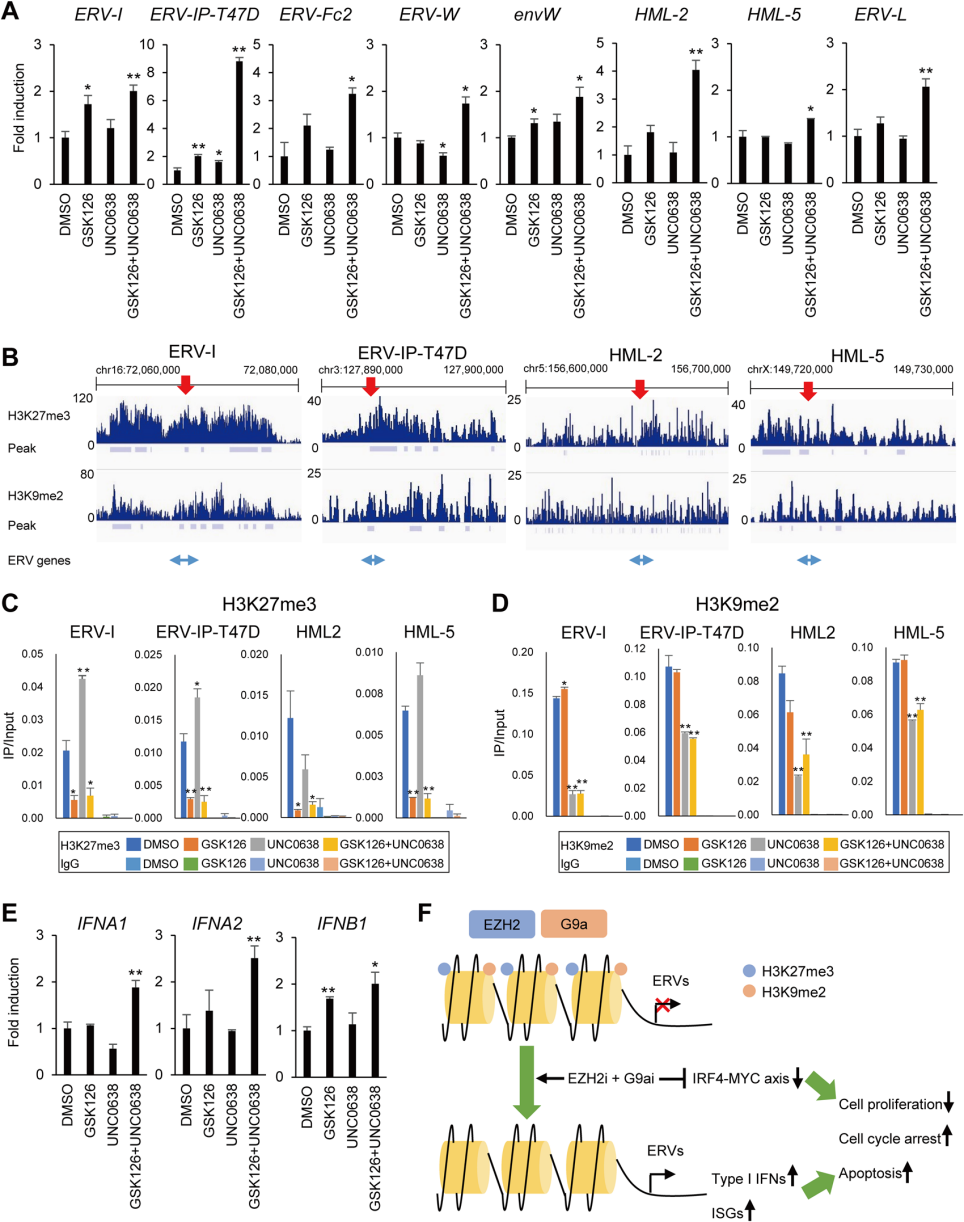
Activation of ERV gene transcription by EZH2 and G9a inhibition in MM cells. **A** qRT-PCR analysis of ERV genes in RPMI-8226 cells treated with the indicated inhibitors (1 μM each, 6 days). Results are normalized to cells treated with DMSO. Shown are means of three technical replications; error bars represent SEMs. **B** ChIP-seq analysis of ERV genes in RPMI-8226 cells. The numbers on the vertical axis indicate the numbers of sequence reads. Regions analyzed by ChIP-PCR are indicated by red arrows on the top, and the locations of ERV genes are indicated at the bottom. Peak, peaks detected by MACS2.0. **C**, **D** ChIP-qPCR analysis showing levels of H3K27me3 (**C**) and H3K9me2 (**D**) at ERV genes in RPMI-8226 cells treated with the indicated inhibitors. Results are normalized to respective input DNAs. Shown are means of three technical replications; error bars represent SEMs. **E** qRT-PCR analysis of interferon genes in RPMI-8226 cells treated with the indicated inhibitors. *P < 0.05, **P < 0.01. **F** Hypothesized mechanism of the antimyeloma effect of EZH2/G9a inhibition.

Recent studies demonstrated that epigenetic drugs activate type I or type III IFN signaling in cancer cells by activating transcription of ERV genes^17,18^. We therefore assessed the expression of ERV genes in MM cell lines treated with EZH2/G9a inhibitors. qRT-PCR analysis revealed that multiple ERV genes were significantly upregulated by GSK126 + UNC0638 in RPMI-8226 and KMS-11 cells, although they were not induced in MM.1S cells (Fig. 5A and Supplementary Fig. S6A). This upregulation of ERV genes was confirmed by treating MM cells with a second set of EZH2/G9a inhibitors, EPZ-6438 and UNC0642 (Supplementary Fig. S7A).

To clarify the mechanism underlying the activation of ERV gene transcription, we assessed histone modifications in MM cells. ChIP-seq and ChIP-qPCR analyses revealed enrichment of H3K27me3 and H3K9me2 in ERV gene regions, while transcriptionally active genes (e.g., *GAPDH* and *ACTB*) were depleted of these modifications (Fig. 5B and Supplementary Fig. S8A–D). Reductions in the levels of H3K27me3 and H3K9me2 in MM cells treated with the EZH2/G9a inhibitors were confirmed by ChIP-qPCR analyses (Fig. 5C, D and Supplementary Fig. S8E, F). This suggests that downregulation of repressive histone modifications may underlie the transcriptional activation of ERV genes in MM cells.

We next analyzed the expression of IFN genes in MM cells treated with the inhibitors. qRT-PCR analysis revealed the dual EZH2/G9a inhibition led to upregulation of *IFNA1*, *IFNA2*, and *IFNB1* (Fig. 5E and Supplementary Figs. S6B, S7B). By contrast, we observed no upregulation of type III IFN genes (*IFNL* family). This suggests that dual EZH2/G9a inhibition downregulates repressive histone marks in ERV genes, which leads to activation of type I IFN signaling in MM cells.

## Discussion

In this study, we found that dual inhibition of EZH2 and G9a exerts a strong antimyeloma effect by upregulating IFN signaling and suppressing the IRF4–MYC axis (Fig. 5F). Recent studies reported that elevated expression of *EZH2* and *EHMT2* is significantly associated with a poor prognosis in MM patients, which indicates that both EZH2 and G9a may be associated with progression of MM^8,19^.

EZH2 is a member of polycomb repressive complex 2 (PRC2) and catalyzes mono-methylation, di-methylation, and tri-methylation at H3K27^6^. Earlier studies showed that inhibition of EZH2 exerts antimyeloma effects through multiple mechanisms, including suppression of oncogenes, activation of tumor suppressor genes, and induction of cell cycle arrest and apoptosis^7–9^. G9a is a histone methyltransferase that catalyzes mono-methylation and di-methylation at H3K9^6^. Several studies have shown the antitumor effects of G9a inhibition in various human malignancies, including breast cancer, acute myeloid leukemia, and non-small cell lung cancer^20–22^. We and others also recently reported the antimyeloma effects of G9a inhibitors^10,19^. Although H3K27 and H3K9 methylation similarly control cellular processes through gene silencing, their functions have been investigated separately in most earlier studies^23,24^. However, more recent studies have begun to reveal that EZH2 and G9a act cooperatively to mediate gene silencing in both normal and cancer cells^13–15^. Our study is the first to show that dual targeting of EZH2 and G9a exerts a strong antitumor effect in MM.

Our transcriptome analysis revealed that dual EZH2/G9a inhibition activated type I IFN signaling in MM cells. IFN-α reportedly exerts an antimyeloma effect by inducing cell cycle arrest and apoptosis^25–28^. In addition, a more recent study showed that activation of STAT1 is required for IFN-α-induced apoptosis in MM cells^29^. IFN-α was first used as monotherapy to treat MM patients in the 1980s, and since the 1990s IFN-α-containing regimens have been used as a central maintenance therapy^30^. The precise mechanism underlying the antimyeloma effects of IFN-α remains unclear, however. A recent study showed that ISG15, an ISG we noted in the present study, induces apoptosis in MM cells^31^. These results suggest that activation of type I IFN signaling contributes to the antitumor effect of EZH2/G9a inhibition in MM.

Chemotherapeutic drugs often exert antitumor effects by inducing immune responses. For instance, by activating Toll-like receptor 3 (TLR3), anthracyclines stimulate production of type I IFNs by cancer cells, and tumors lacking TLR3 or IFN-α receptor fail to respond to anthracycline chemotherapy^32^. This suggests that “viral mimicry” mediated by anthracycline is essential for successful chemotherapy. The antitumor effects of DNMT inhibitors such as 5-azacytidine and 5-aza-2′-deoxycytidine are also associated with activation of IFN signaling^33^. DNMT inhibitors induce viral mimicry by activating ERV gene transcription in cancer cells^17,18,34^. Double-stranded RNAs (dsRNAs) derived from sense and antisense ERV genes transcripts activate cytosolic dsRNA sensors, including TLR3 and MAVS, causing type I or type III IFN responses. Moreover, another study showed that dual inhibition of DNMT and G9a induces upregulation of ERV gene expression and viral mimicry in ovarian cancer cells^35^. This suggests that methylation of both DNA and H3K9 is involved in the repression of ERV genes. Notably, in a murine MM model, a combination of DNMT and HDAC inhibitors also upregulated expression of an ERV gene and activated type I IFN signaling in tumor cells^36^. Taken together with those reports, our findings suggest that activation of type I IFN signaling through dual EZH2/G9a inhibition is due, at least in part, to activation of ERV gene transcription in MM.

A number of studies have demonstrated that EZH2 and G9a are involved in the regulation of immune responses in both normal and cancer cells. For instance, PRC2 represses hundreds of ISGs, cytokines, and cytokine receptors in cancer cells, while EZH2 inhibitors activate PRC2-repressed immune genes^37^. EZH2 negatively regulates mitochondria-mediated antiviral innate immune responses by blocking the RIG-I/MAVS RNA recognition pathway, and inhibition of EZH2 activates infection-induced IFN-β expression^38^. EZH2 inhibitors, including GSK126, reportedly induce a cellular antiviral state through upregulation of IFN-α and ISGs, and suppress infection by DNA and RNA viruses^39^. Moreover, SPARCS (stimulated three prime antisense retroviral coding sequences) were recently identified as a novel subclass of ERVs silenced by EZH2^40^. SPARCS are located in the antisense of the 3′ untranslated regions of IFN-stimulated genes, and low levels of EZH2 trigger expression of these ERVs when exposed to IFN-γ, leading to innate immune signaling in cancer. G9a and H3K9 methylation also suppresses expression of IFNs and ISGs, which leads to cell type-specific differences in IFN signaling. In mouse fibroblasts, G9a silences Ifnb1 and IFN-inducible gene expression, while G9a inhibition enhances resistance to viral infection^41^. In bladder cancer, high *EHMT2* expression is associated with poor clinical outcomes, and a novel dual G9a/DNMT inhibitor, CM-272, reportedly induces apoptosis and immunogenic cell death^42^. In the present study, dual targeting of EZH2 and G9a significantly affected expression of immune-related genes in MM cells, including IFNs and ISGs. The upregulated expression and decreased repressive histone modifications of multiple ERV genes suggest that derepression of ERVs may trigger IFN signaling in MM. However, although upregulation of ERVs through EZH2/G9a inhibition was observed in multiple MM cell lines (RPMI-8226 and KMS-11), expression of the ERVs tested remained unchanged in MM.1S cells. This suggests mechanisms other than de-repression of ERVs are also involved in the stimulation of IFN signaling in MM cells by EZH2/G9a inhibition.

We also found that inhibiting EZH2 and G9a downregulates important oncogenes, including *IRF4*, *MYC*, *KLF2*, and *PRDM1*, in MM cells. Although earlier studies showed that EZH2 inhibition reduces expression of *IRF4*, *MYC*, and *PRDM1* in MM, we found that the dual EZH2/G9a inhibition exerted greater suppressive effects than inhibition of either enzyme alone^8,9^. Survival of MM cells is strongly dependent on the IRF4–MYC axis, within which IRF4 and MYC reciprocally transactivate each other, generating an autoregulatory circuit in MM cells^16^. In addition, the KDM3A–KLF2–IRF4 axis also contributes to MM cell survival^43^. We previously showed that DOT1L inhibition leads to decreased H3K79 methylation and reduced expression of IRF4–MYC axis genes in MM^10^. Our present results suggest that targeting EZH2/G9a also blocks MM cell proliferation by suppressing this axis. However, further study will be necessary to clarify the mechanism by which EZH2/G9a inhibition leads to downregulation of these oncogenes.

In summary, we have shown that dual targeting of EZH2 and G9a is a potentially effective strategy for treating MM. The efficacies of the EZH2 inhibitors EPZ-6438 (also known as Tazemetostat) and GSK126 against various cancers, including B-cell lymphoma, have been tested in clinical trials^5,12^. By contrast, no G9a inhibitor is currently in clinical trials. Thus, development of a novel G9a inhibitor or a dual inhibitor of both EZH2 and G9a would be desirable. This study is the first to demonstrate a relationship between histone methylation and immune responses in MM. Recent studies have shown that epigenetic drugs such as DNMT and HDAC inhibitors can sensitize cancer cells to immune checkpoint inhibitors^17,44^. Further studies to clarify the clinical usefulness of the combination of histone methyltransferase inhibitors and immunomodulatory drugs (IMiDs) or immune checkpoint inhibitors in MM are warranted.

## Materials and methods

### Cell lines and reagents

MM cell lines (RPMI-8226, MM.1S, KMS-11, KMS-12BM, KMS-12PE, and U-266) were obtained and cultured as described previously^10^. Cell lines were authenticated using short tandem repeat analysis performed by JCRB (Tokyo, Japan) or BEX (Tokyo, Japan). They were also checked for mycoplasma with an EZ-PCR Mycoplasma Detection Kit (Biological Industries, Beit HaEmek, Israel) and were found to be negative. Total RNA was extracted using RNeasy Mini Kits (Qiagen, Hilden, Germany) according to the manufacturer’s instructions. EZH2 inhibitors (GSK126 and EPZ-6438) were purchased from Chemietek (Indianapolis, IN, USA). G9a inhibitors (UNC0638 and UNC0642) were from Sigma-Aldrich (St. Louis, MO, USA).

### Drug treatment and cell viability assays

To assess the antiproliferative effects of the EZH2 and G9a inhibitors, MM cell lines (2 × 10^4^ to 1 × 10^5^ cells/well in 6-well plate) were treated with a single inhibitor (1 μM) or a combination of the two inhibitors (1 μM each) or with DMSO for up to 14 days, refreshing the medium and drugs every 3 days. Cell viability was assessed on days 3, 6, and 14 using a Cell Counting Kit-8 (Dojindo, Kumamoto, Japan) and a microplate reader (Model 680; Bio-Rad, Hercules, CA, USA) according to the manufacturer’s instructions.

### Xenograft studies

For xenograft studies, we used the ex vivo drug pre-treatment method^10^. RPMI-8226 cells were pretreated for 24 h with 1 μM GSK126 plus 1 μM UNC0638 or with DMSO, after which 1 × 10^7^ cells were suspended in 200 μl of RPMI-1640 medium and subcutaneously injected into the bilateral thighs of 6-week-old C.B-17 SCID female mice. A sample size of 5 was chosen for the xenograft study. No randomization was used, and the researchers were not blinded to the experiments. Tumor size was measured every 3 days using digital calipers, and tumor volume was calculated using the formula, length × width^2^/2. All animal experiments were conducted in compliance with a protocol approved by the Institutional Animal Care and Use Committee of Sapporo Medical University.

### Quantitative reverse transcription-PCR

Single-strand cDNA was prepared using PrimeScript RT Master Mix (Takara, Tokyo, Japan), after which the integrity of the cDNA was confirmed by amplifying β-actin (*ACTB*). Quantitative reverse transcription-PCR (qRT-PCR) was carried out using Power Up SYBR Green Master Mix (Thermo Fisher Scientific, Waltham, MA, USA) and a 7500 Fast Real-Time PCR System (Thermo Fisher Scientific). Primer sequences and PCR product sizes are listed in Supplementary Table S1. Primers for endogenous retrovirus (ERV) genes were as described^45–47^.

### Western blot analysis

Total proteins were extracted using Cell Lysis Buffer (#9803, Cell Signaling Technology, Danvers, MA, USA) according to the manufacturer’s instructions. Histones were extracted using Triton Extraction Buffer (TEB) according to the protocol from Abcam (Cambridge, UK). Samples were separated using SDS-PAGE (12% acrylamide) and transferred to PVDF membranes (BioRad). The membranes were then blocked using TBST with 5% bovine serum albumin or Block Ace (KAC Co., Ltd., Kyoto, Japan) and incubated overnight with rabbit anti-Stat1 mAb (1:1000 dilution, #14994; Cell Signaling Technology), rabbit anti-phospho-Stat1 mAb (1:1000 dilution, #9167; Cell Signaling Technology), rabbit anti-Ezh2 mAb (1:1000 dilution, #5246; Cell Signaling Technology), rabbit anti-G9a/EHMT2 mAb (1:1000 dilution, #68851; Cell Signaling Technology), mouse anti-β-actin Ab (1:2000 dilution, #A5441; Sigma-Aldrich), mouse anti-mono-methyl histone H3K27 mAb (1:455 dilution, #61015; Active Motif Japan, Tokyo, Japan), rabbit anti-di-methyl histone H3K27 mAb (1:1000 dilution, #9728; Cell Signaling Technology), rabbit anti-tri-methyl histone H3K27 mAb (1:1000 dilution, #9733; Cell Signaling Technology), rabbit anti-mono-methyl histone H3K9 mAb (1:1000 dilution, #14186; Cell Signaling Technology), rabbit anti-di-methyl histone H3K9 mAb (1:1000 dilution, #4658; Cell Signaling Technology), rabbit anti-tri-methyl histone H3K9 mAb (1:1000 dilution, #13969; Cell Signaling Technology), or rabbit anti-H3 mAb (1:2000 dilution, #4499; Cell Signaling Technology). Signals were detected using HRP-conjugated secondary antibodies (Cell Signaling Technology). Luminescent signals were detected using an ImageQuant LAS-4000 mini image reader (GE Healthcare Japan, Hino, Japan).

### Flow cytometric analysis

MM cells were treated for 6 days with the EZH2 inhibitor GSK126 (1 μM), the G9a inhibitor UNC0638 (1 μM), a combination of the two, or DMSO as described above, after which cells were stained with propidium iodide (Dojindo) and a ApoScreen Annexin V Apoptosis Kit (Southern Biotech, Birmingham, AL, USA) according to the manufacturer’s instructions. Flow cytometric analysis was then performed using a BD FACSCant II (BD Biosciences, Franklin Lakes, NJ, USA) with BD FACSDiva software (BD Biosciences). Data were analyzed using FlowJo software version 10 (FlowJo LLC, Ashland, OR, USA).

### Gene expression microarray analysis

Gene expression was analyzed as described previously^10^. Briefly, 100 ng of total RNA were amplified and labeled using a Low-input Quick Amp Labeling kit One-color (Agilent Technologies, Santa Clara, CA, USA). The synthesized cRNA was hybridized to a SurePrint G3 Human GE microarray v2 (G4858A #39494; Agilent Technologies). The microarray data were then imported into Gene Spring GX version 14 (Agilent Technologies). Gene ontology and pathway analyses were also performed using Gene Spring GX. Gene set enrichment analysis (GSEA) was performed using a gene list for IFN-α/β signaling provided by WikiPathways (WP1835_101367). The Gene Expression Omnibus accession number for the microarray data is GSE155135.

### Chromatin immunoprecipitation sequencing and quantitative PCR

Chromatin immunoprecipitation (ChIP) was performed as described previously^10^. Briefly, 1 × 10^6^ cells were treated for 10 min with 0.5% formaldehyde. After washing, the cells were resuspended in 110 μL of lysis buffer and sonicated. Chromatin was immunoprecipitated for 12 h at 4 °C using 4 μL of rabbit anti-trimethyl histone H3K27 mAb (#9733, Cell Signaling Technology), 0.1 μL of rabbit (DA1E) mAb IgG XP isotype control (#3900, Cell Signaling Technology), 2.5 μL of mouse anti-di-methyl histone H3K9 mAb (#1220, Abcam) or 1 μL of mouse (G3A1) mAb IgG1 isotype control (#5415, Cell Signaling Technology). Before adding the antibody, 8 μL of each cell lysate was saved as an internal control for the input DNA. After washing, elution, reversal of the cross-links and DNA purification, ChIP sequencing (ChIP-seq) and quantitative PCR (ChIP-qPCR) were performed. For ChIP-seq, samples were prepared using a SMARTer ThruPLEX DNA-seq Kit (Takara) according to the manufacturer’s instructions and were sequenced using a NextSeq 550 system (Illumina, San Diego, CA, USA). Data analysis and ChIP-qPCR were carried out as described^10^. Locations of the ERV genes were identified by using the gEVE database (http://geve.med.u-tokai.ac.jp)^48^. Primers used in the qRT-PCR analysis of ERV genes were also used in the ChIP-qPCR analysis.

### Statistical analysis

To analyze EZH2 or G9a expression in clinical samples, published datasets (GSE5900, GSE6477, and GSE4581) were obtained from the Gene Expression Omnibus. Expression levels were analyzed using Student’s *t*-test. Kaplan–Meier curves were plotted to compare survival in two groups stratified based on expression levels of *EZH2* or *EHMT2*. Comparisons between groups were made using the log-rank test. Results of cell viability assays, qRT-PCR and ChIP-qPCR were analyzed using Student’s *t*-test or one-way ANOVA. Each analyzed dataset was derived from at least three independent experiments because statistical significance was observed using the indicated sample sizes. Values of *P* < 0.05 (two-sided) were considered significant. Data were analyzed using EZR version 1.32 (Saitama Medical Center, Jichi Medical University, Saitama, Japan).

## Supplementary information

Supplementary Figure legends

Supplementary Figure legends

Supplementary Figure 1

Supplementary Figure 2

Supplementary Figure 3

Supplementary Figure 4

Supplementary Figure 5

Supplementary Figure 6

Supplementary Figure 7

Supplementary Figure 8

Supplementary Table legends

Supplementary Table 1

Supplementary Table 2

Supplementary Table 3

Supplementary Table 4

Supplementary Table 5

Supplementary Table 6

Supplementary Table 7

Supplementary Table 8

Supplementary Table 9

Supplementary Table 10

Supplementary Table 11

Supplementary Table 12

Supplementary Table 13
