# Supplementary material for: Dual EZH2 and G9a inhibition suppresses multiple myeloma cell proliferation by regulating the interferon signal and IRF4-MYC axis
Source: Cell Death Discov. 2021 Jan 12;7:7. doi: 10.1038/s41420-020-00400-0 (PMC7803977; doi:10.1038/s41420-020-00400-0)
Supplement: Supplementary file 1 — Supplementary Figure legends [file 41420_2020_400_MOESM1_ESM.docx]

**Supplementary Figure legends**

**Supplementary Figure 1**

Western blot analysis showing levels of histone H3K9 and H3K27 methylation in sensitive (A) and resistant (B) multiple myeloma (MM) cell lines treated with a EZH2 inhibitor (GSK126, 1 μM, 6 days ) and/or a G9a inhibitor (UNC0638, 1 μM, 6 days). Histone H3 is shown as a loading control.

**Supplementary Figure 2**

Western blot analysis showing levels of Ezh2 and G9a in multiple myeloma (MM) cell lines. β-actin is shown as a loading control.

**Supplementary Figure 3**

Microarray analysis of gene expression in MM cells after EZH2 and G9a inhibition. Shown are Venn diagrams of genes whose expression was up- or downregulated (> 2-fold) by the indicated drugs in the respective MM cell lines.

**Supplementary Figure 4**

qRT-PCR analysis of interferon-stimulated genes (ISGs) in MM.1S cells treated with an EZH2 inhibitor (EZP-6438, 1 μM, 6 days) and/or a G9a inhibitor (UNC0642, 1 μM, 6 days). Results are normalized to cells treated with DMSO. Shown are means of 3 technical replications; error bars represent standard errors of means (SEMs). **P*<0.05. ***P*<0.01.

**Supplementary Figure 5**

qRT-PCR analysis of IRF4-MYC axis genes in MM cell lines treated with the indicated inhibitors (1 μM each, 6 days). Results are normalized to cells treated with DMSO. Shown are means of 3 technical replications; error bars represent SEMs. **P*<0.05. ***P*<0.01.

**Supplementary Figure 6**

Upregulation of ERV and interferon genes in MM cells. (A,B) qRT-PCR analysis of ERV (A) and interferon genes (B) in KMS-11 cells treated with the indicated inhibitors (1 μM, 6 days). Results are normalized to cells treated with DMSO. Shown are means of 3 technical replications; error bars represent SEMs. **P*<0.05. ***P*<0.01.

**Supplementary Figure 7**

Upregulation of ERV and interferon genes by treatment with EPZ-5676 and UNC0642 in MM cells. (A,B) qRT-PCR analysis of ERV (A) and interferon genes (B) in RPMI-8226 cells treated with the indicated inhibitors (1 μM, 6 days). Results are normalized to cells treated with DMSO. Shown are means of 3 technical replications; error bars represent SEMs. **P*<0.05. ***P*<0.01.

**Supplementary Figure 8**

Activation of ERV gene transcription by EZH2 and G9a inhibition in MM cells. (A) ChIP-seq analysis of ERV genes in RPMI-8226 cells. The numbers on the vertical axis indicate the numbers of sequence reads. Regions analyzed by ChIP-PCR are indicated by red arrows on the top and locations of ERV genes are indicated at the bottom. Peak, peaks detected by MACS2.0. (B) ChIP-seq analysis of the housekeeping genes ACTB and GAPDH. Regions analyzed by ChIP-PCR are indicated by red arrows on the top. TSS, transcription start site. (C, D) ChIP-qPCR analysis showing levels of H3K27me3 (C) and H3K9me2 (D) at the ERV genes and housekeeping genes (ACTB and GAPDH) in RPMI 8226 cells. Results are normalized to the respective input DNAs. Shown are means of 3 technical replications; error bars represent SEMs. (E, F) ChIP-qPCR analysis showing levels of H3K27me3 (E) and H3K9me2 (F) at the ERV genes in RPMI-8226 cells treated with the indicated inhibitors. Results are normalized to respective input DNAs. Shown are means of 3 technical replications; error bars represent SEMs. **P*<0.05. ***P*<0.01.
