# Supplementary figures and images for: Dual EZH2 and G9a inhibition suppresses multiple myeloma cell proliferation by regulating the interferon signal and IRF4-MYC axis
Source: Cell Death Discov. 2021 Jan 12;7:7. doi: 10.1038/s41420-020-00400-0 (PMC7803977; doi:10.1038/s41420-020-00400-0)

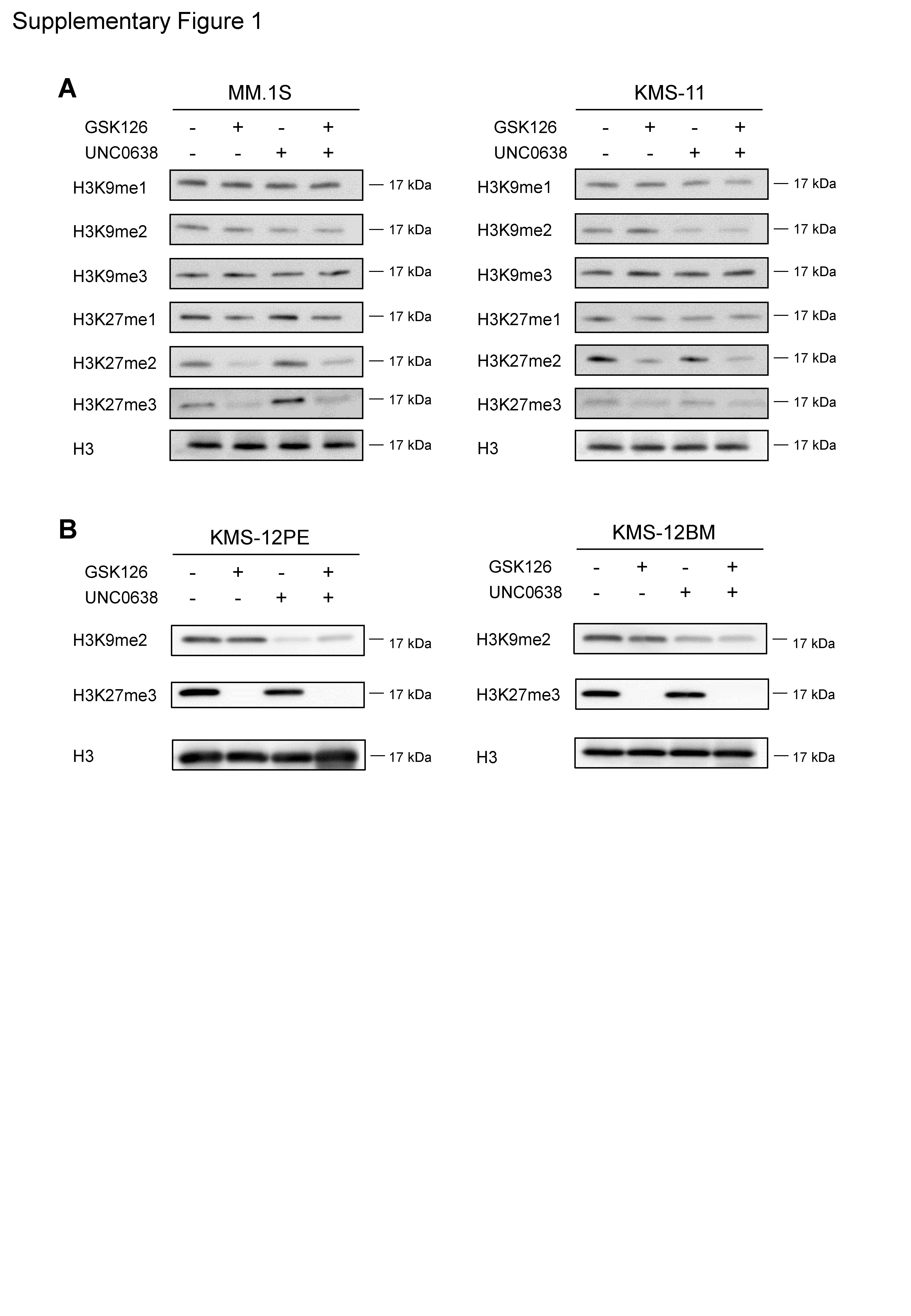

Supplement: Supplementary file 3 — Supplementary Figure 1 [file 41420_2020_400_MOESM3_ESM.png]

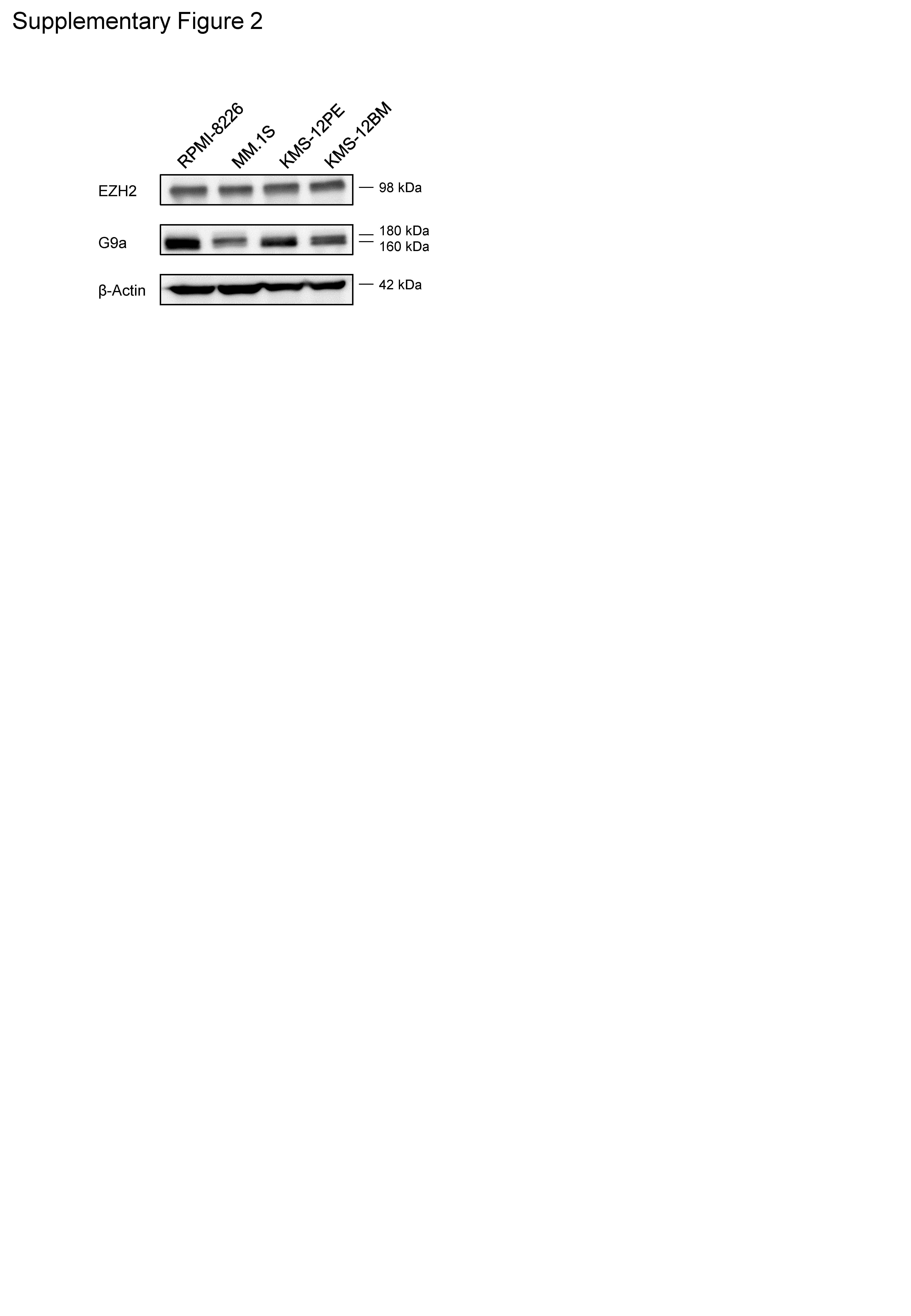

Supplement: Supplementary file 4 — Supplementary Figure 2 [file 41420_2020_400_MOESM4_ESM.png]

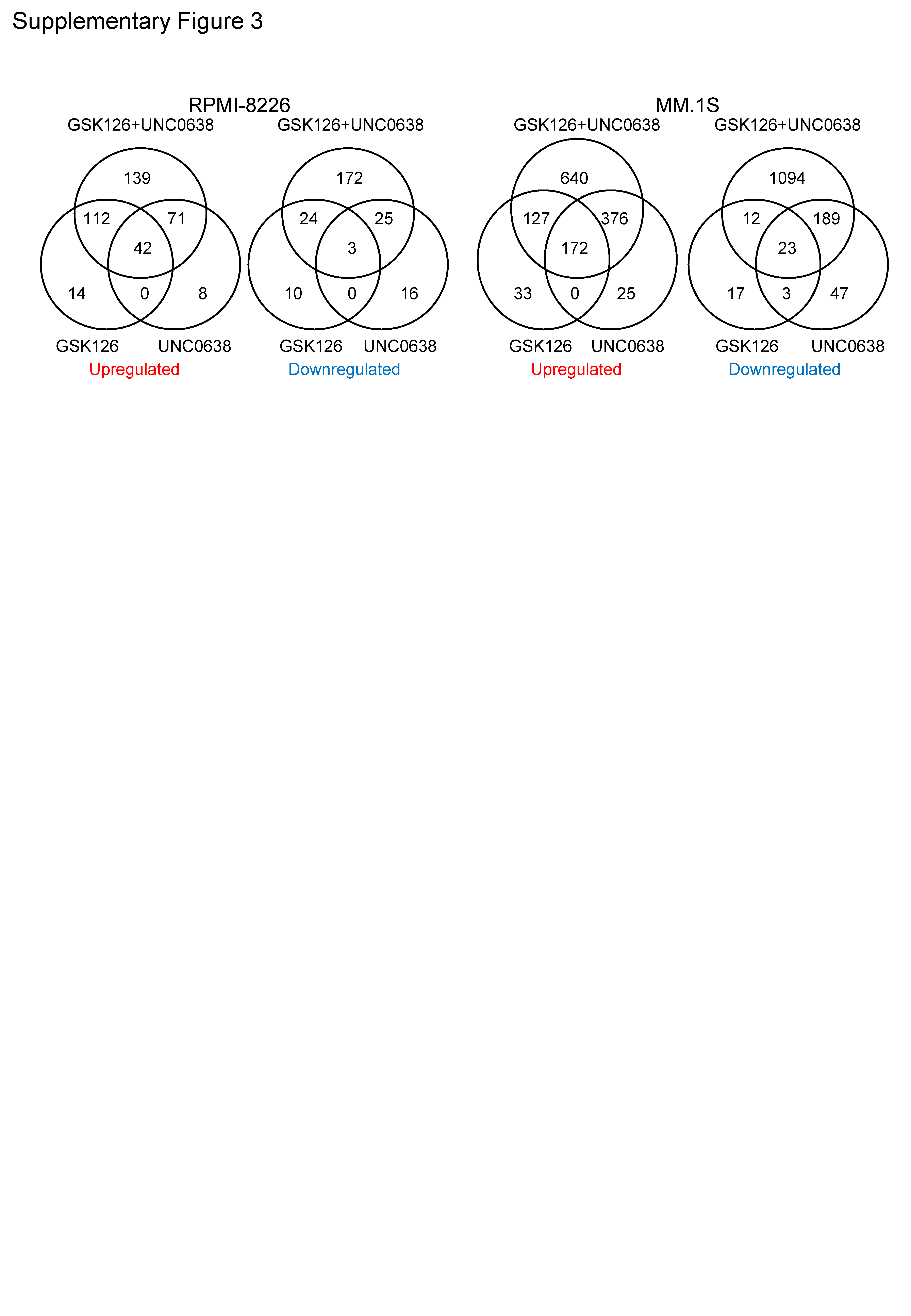

Supplement: Supplementary file 5 — Supplementary Figure 3 [file 41420_2020_400_MOESM5_ESM.png]

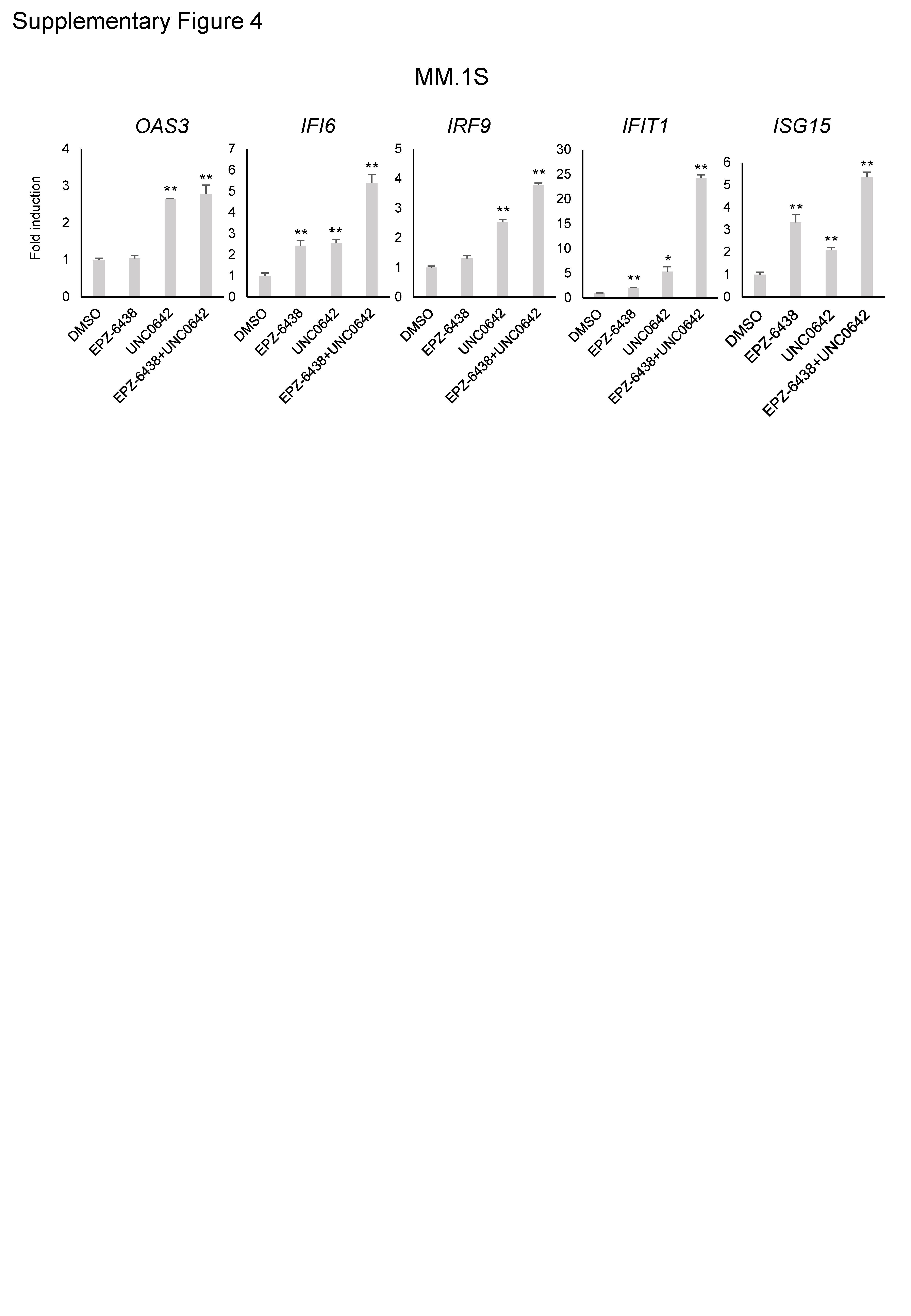

Supplement: Supplementary file 6 — Supplementary Figure 4 [file 41420_2020_400_MOESM6_ESM.png]

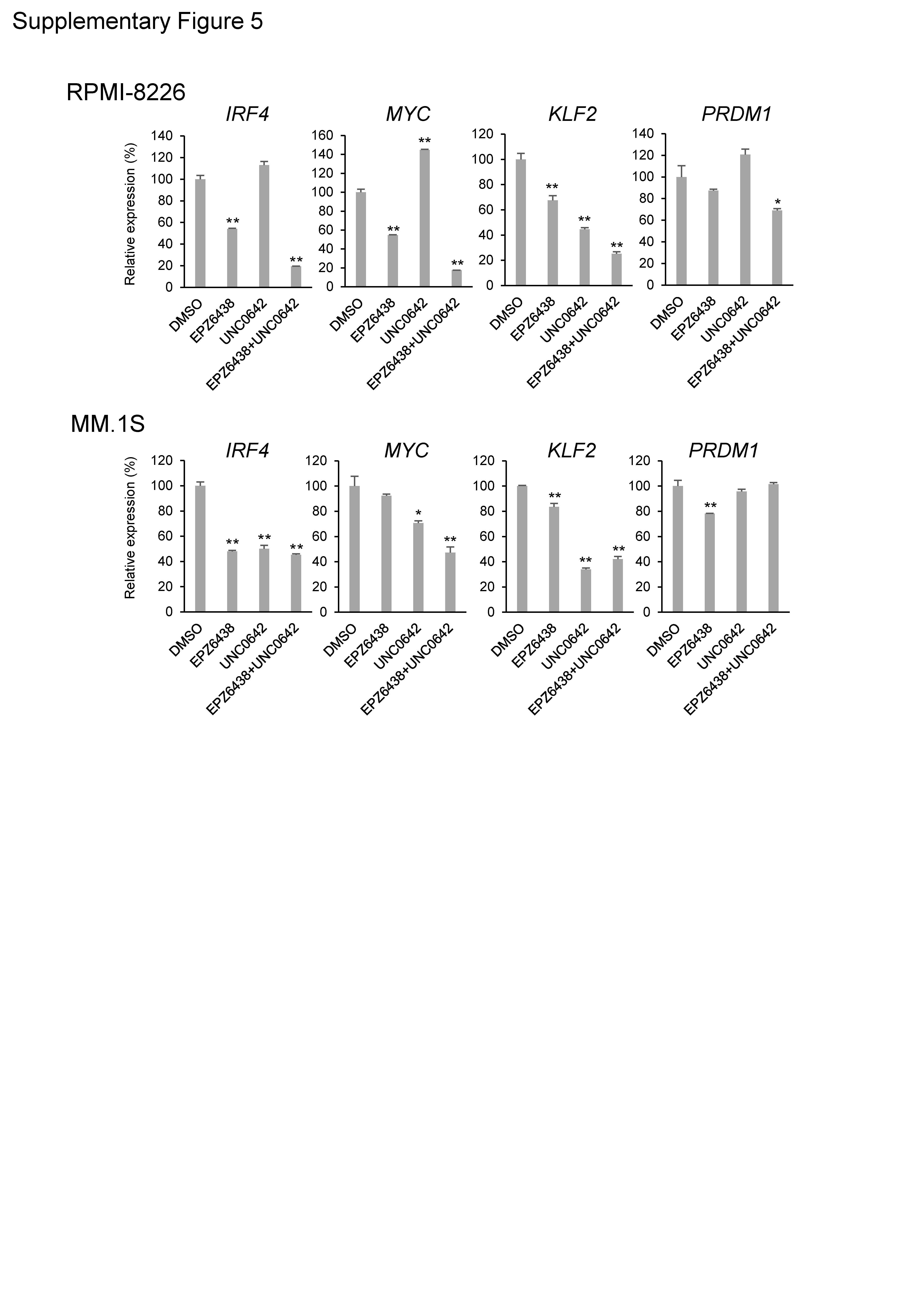

Supplement: Supplementary file 7 — Supplementary Figure 5 [file 41420_2020_400_MOESM7_ESM.png]

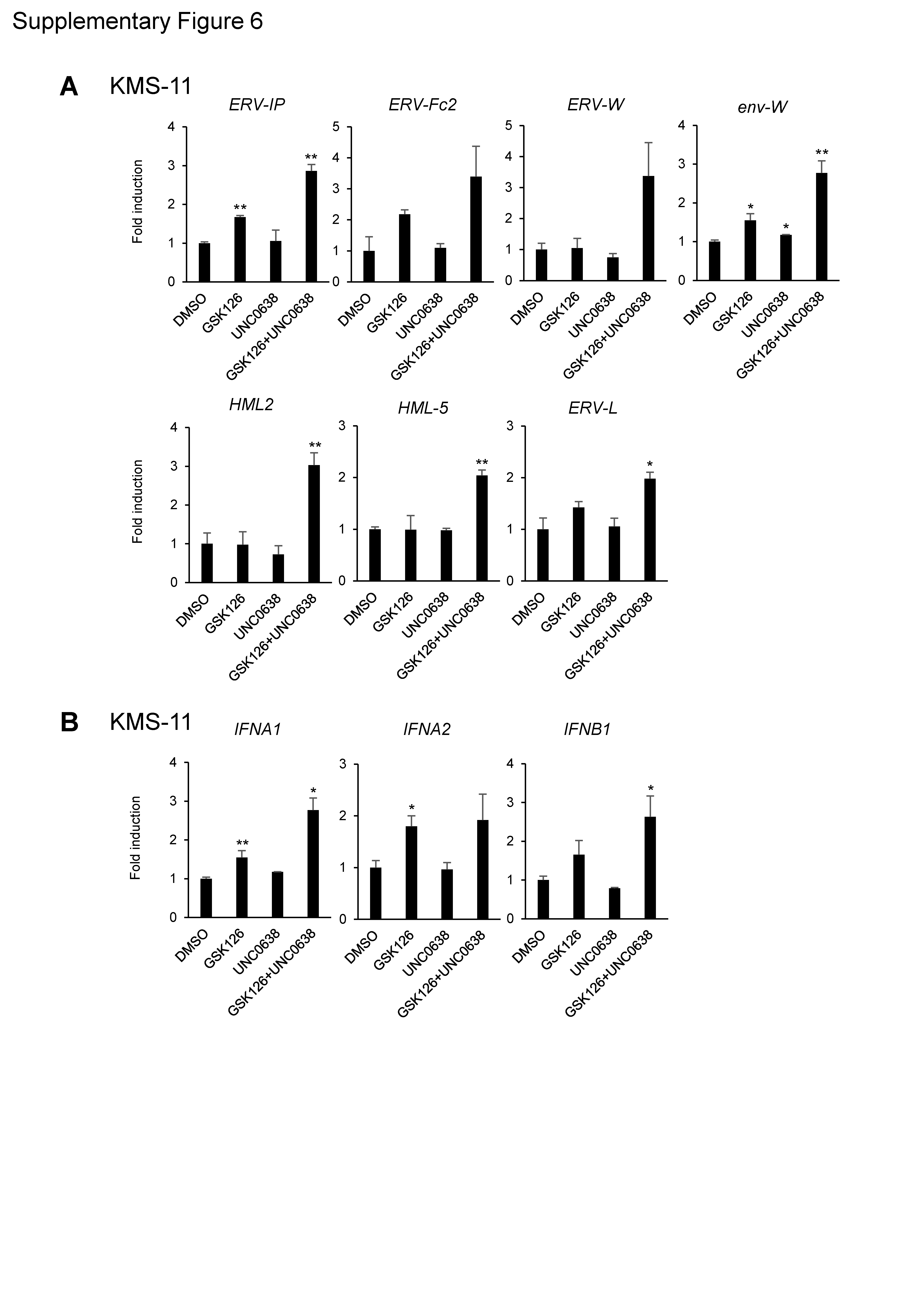

Supplement: Supplementary file 8 — Supplementary Figure 6 [file 41420_2020_400_MOESM8_ESM.png]

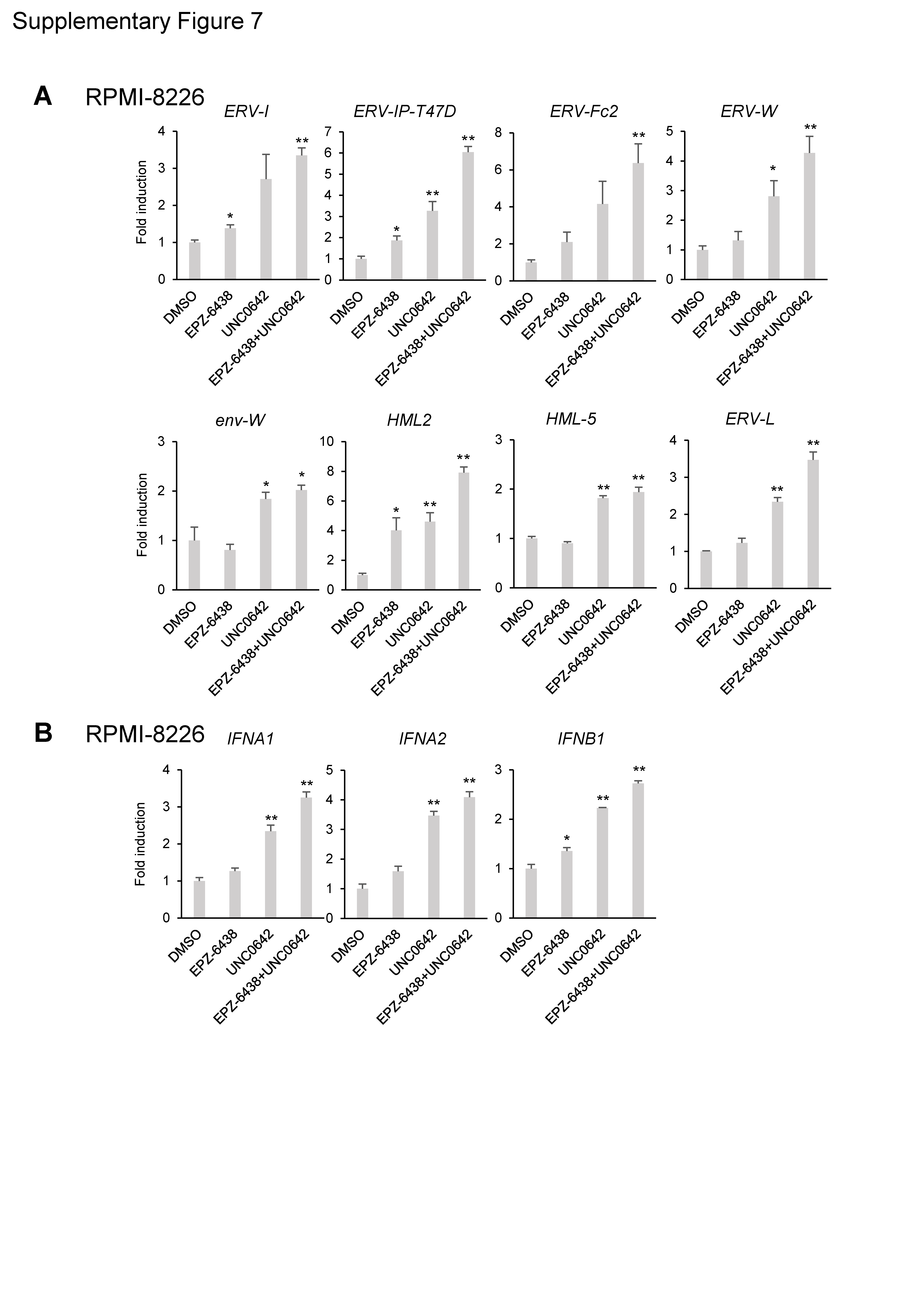

Supplement: Supplementary file 9 — Supplementary Figure 7 [file 41420_2020_400_MOESM9_ESM.png]

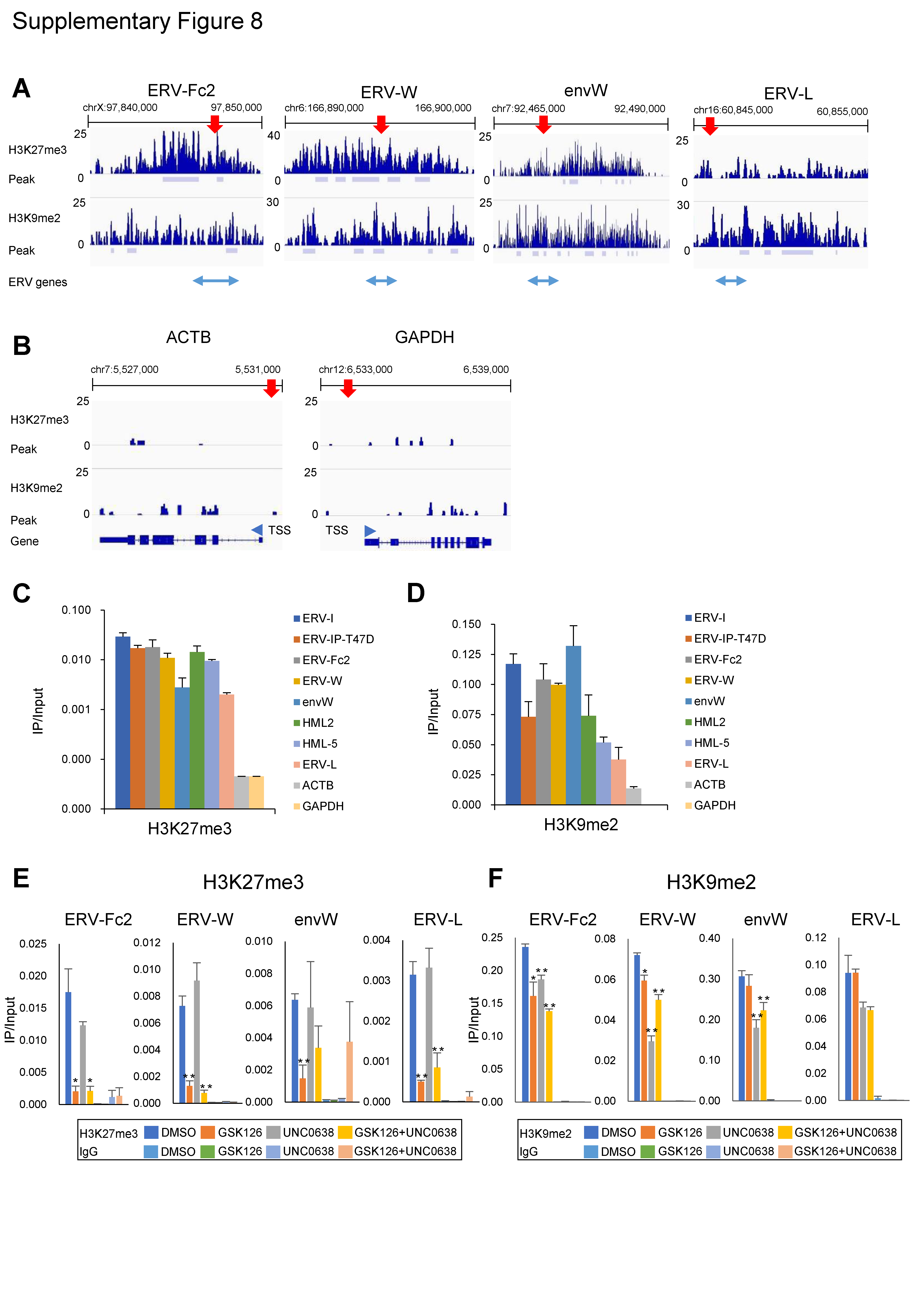

Supplement: Supplementary file 10 — Supplementary Figure 8 [file 41420_2020_400_MOESM10_ESM.png]
