# Supplementary material for: Dual EZH2 and G9a inhibition suppresses multiple myeloma cell proliferation by regulating the interferon signal and IRF4-MYC axis
Source: Cell Death Discov. 2021 Jan 12;7:7. doi: 10.1038/s41420-020-00400-0 (PMC7803977; doi:10.1038/s41420-020-00400-0)
Supplement: Supplementary file 11 — Supplementary Table legends [file 41420_2020_400_MOESM11_ESM.docx]

**Supplementary Table legends**

**Supplementary Table 1**

Sequences of the primers used in this study.

**Supplementary Table 2**

Genes whose expression was upregulated by GSK126 in RPMI-8226 cells.

**Supplementary Table 3**

Genes whose expression was upregulated by UNC0638 in RPMI-8226 cells.

**Supplementary Table 4**

Genes whose expression was upregulated by GSK126 + UNC0638 in RPMI-8226 cells.

**Supplementary Table 5**

Genes whose expression was downregulated by GSK126 in RPMI-8226 cells.

**Supplementary Table 6**

Genes whose expression was downregulated by UNC0638 in RPMI-8226 cells.

**Supplementary Table 7**

Genes whose expression was downregulated by GSK126 + UNC0638 in RPMI-8226 cells.

**Supplementary Table 8**

Genes whose expression was upregulated by GSK126 in MM.1S cells.

**Supplementary Table 9**

Genes whose expression was upregulated by UNC0638 in MM.1S cells.

**Supplementary Table 10**

Genes whose expression was upregulated by GSK126 + UNC0638 in MM.1S cells.

**Supplementary Table 11**

Genes whose expression was downregulated by GSK126 in MM.1S cells.

**Supplementary Table 12**

Genes whose expression was downregulated by UNC0638 in MM.1S cells.

**Supplementary Table 13**

Genes whose expression was downregulated by GSK126 + UNC0638 in MM.1S cells.
